# Impact of peri-operative blood transfusion on post-operative infections after radical gastrectomy for gastric cancer: a propensity score matching analysis focusing on the timing, amount of transfusion and role of leukocyte depletion

**DOI:** 10.1007/s00432-018-2630-8

**Published:** 2018-03-23

**Authors:** Hua Xiao, Hu Quan, Shuguang Pan, Bin Yin, Wei Luo, Gang Huang, Yongzhong Ouyang

**Affiliations:** Department of Gastroduodenal and Pancreatic Surgery, Hunan Cancer Hospital and the Affiliated Cancer Hospital of Xiangya School of Medicine, Central South University, 283 Tongzipo Road, Changsha, 410013 Hunan China

**Keywords:** Gastric cancer, Gastrectomy, Transfusion, Infection

## Abstract

**Purpose:**

Allogeneic blood transfusions (BTF) are sometimes inevitable during radical gastrectomy with lymphadenectomy for advanced gastric cancer. The aim of this retrospective study was to investigate the association between BTF and post-operative infections, focusing on the impact of timing, amount of transfusion and the role of leukocyte depletion.

**Methods:**

The study cohort was 2064 patients who underwent gastrectomy for gastric cancer from November 2010 to August 2017. The association between BTF and post-operative infections was estimated by univariate and multivariate analyses after propensity score matching. Subgroup analysis was performed according to the timing and amount of transfusion, and leukocyte depletion or not.

**Results:**

Out of a total 2064 patients, 426 (20.6%) received peri-operative BTF. After one-to-one matching, 361 pairs of patients were included for further analysis, of who 68 (9.4%) developed infections. Multivariate analysis identified that an operation time ≥ 240 min, combined multi-organ resection, BTF and BMI ≥ 25 kg/m^2^ were independent risk factors for post-operative infection. Patients given a high-volume (> 7.5 U), intra-operatively of leukocyte-non-depleted BTF had the highest risk of developing infections clarified by subgroup analysis.

**Conclusion:**

Infection was the most common complication following gastrectomy for gastric cancer and BTF was identified as an independent risk factor by propensity score matching and multivariate analyses. The timing, amount of transfusion and leukocyte depletion had an impact on the incidence of infection. To decrease infection, BTF should be avoided where possible, particularly during operation, with a large amount and leukocyte-not-depleted blood.

## Introduction

Gastric cancer is the fourth most frequent cancer occurring worldwide and the second most common cause of cancer-related mortality in China (Torre et al. 2015; Chen et al. 2016), with to date surgery as the only treatment that offers a curative result. Unfortunately, the overwhelming majority of patients in China and Western countries are diagnosed at an advanced stage and radical gastrectomy with D2 lymph node dissection is recommended in the guidelines for these patients in the East and West (Japanese Gastric Cancer Association 2017; Ajani et al. 2016; Smyth et al. 2016). A high frequency of patients with advanced gastric cancer present with anemia, and furthermore, gastrectomy with lymphadenectomy sometimes causes excessive bleeding even when performed by experienced surgeons (Birgegård et al. 2005; Sasako et al. 2008). Thus, allogeneic blood transfusion (BTF) is sometimes inevitable when performing D2 gastrectomy for advanced gastric cancer, although it is fair to say that the frequency of BTF is decreasing as a result of improvements in surgical techniques and peri-operative care (Ecker et al. 2016). While BTF may be vital in some circumstances, there is growing evidence that BTF is associated with adverse long-term survival in oncological patients. These detrimental effects are thought to be associated with systemic inflammation and transfusion-related immunomodulation (TRIM) (Sun et al. 2015; Squires et al. 2015; Kanda et al. 2016; Aquina et al. 2017), while the relationships between BTF and post-operative short-term outcomes have been less well documented. Most of the current literature that reported investigations into the association between BTF and infection was usually based on a limited number of patients and none of these studies used propensity score-matching analysis to adjust for patient background data such as comorbidities, body mass index (BMI) or extension of resection, which are well-known risk factors for post-operative infection (Bellantone et al. 1998; Jung et al. 2013; Elmi et al. 2016). In addition, the association of the transfusion timing, amount and the role of leukocyte depletion with post-operative infection that focused on patients who underwent gastrectomy for gastric cancer has rarely been studied. Therefore, the present inquiry aimed to evaluate the potential impact of the timing, amount of transfusion and leukocyte depletion on post-operative infection after radical gastrectomy for gastric cancer by a propensity score-matching analysis using the database from a high volume center in China.

## Methods

### Design and patients

**Fig. 1:**
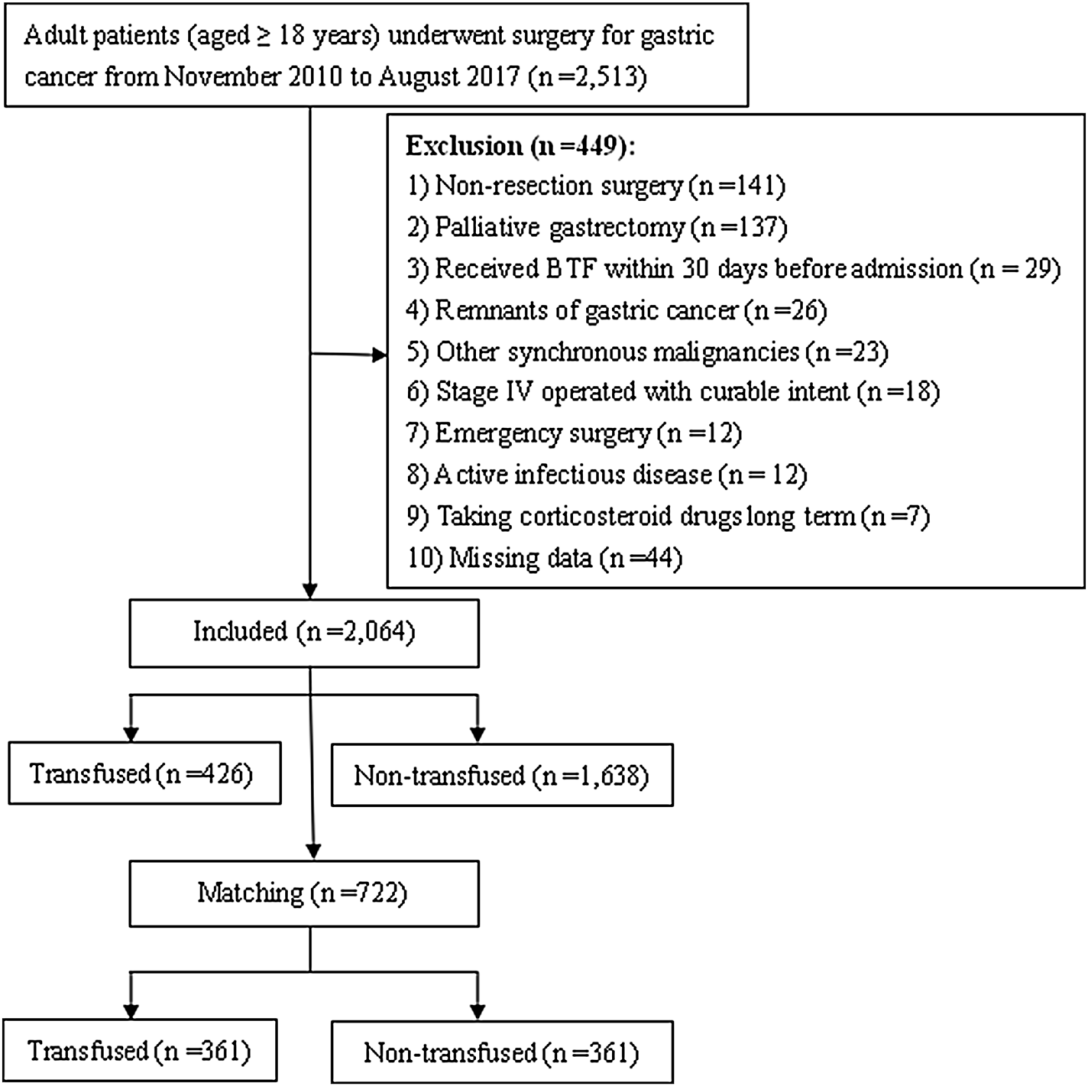
Flow-chart

All consecutive adult patients (≥ 18 years) who underwent surgery with a pathological diagnosed gastric adenocarcinoma between November 1, 2010 and August 31, 2017 in our hospital were screened for inclusion. Exclusion criteria are described in Fig. 1. In total, data from 2,064 patients were analyzed in this retrospective study. Patients were categorized according to whether they received peri-operative BTF or not. The ethics committee of the Affiliated Cancer Hospital of Xiangya School of Medicine, Central South University, approved this study and waived the need for informed consent considering the observational nature of the study design.

### Surgical procedures and peri-operative management

Surgeons with sufficient experience of D2 or D2 + radical gastrectomy performed all operations. Each tumor was graded according to the 7th UICC (Union for International Cancer Control) TNM (Tumor-Lymph Node-Metastasis) Staging System of Gastric Cancer (Kwon 2011). Lymphadenectomy and gastric reconstruction were determined in accordance with Japanese gastric cancer treatment guidelines (Japanese Gastric Cancer Association 2017). The main surgical procedures and peri-operative management have been described in our previous study (Xiao et al. 2017). To be brief, open procedure with D2 or D2 + lymph node dissection was the main surgical type for patients with advanced gastric cancer. Combined multi-organ resection was performed in patients with a locally advanced tumor suspected of invading adjacent organs for the purpose of R0 resection or simultaneous resection of other organs because of benign disease. A 6-mm silicon drain tube was placed in the Morrison pouch and sub-hepatic space, and another placed in the splenic fossa if radical total gastrectomy or combined distal pancreatectomy and splenectomy was performed. A prophylactic antibiotic of a second- or third-generation cephalosporin was administered to all patients and usually lasted for 3–5 days following the operation. Neo-adjuvant chemotherapy was applied in a few patients with advanced gastric cancer, in a standard manner with S-1 plus oxaliplatin (SOX), or epirubicin, cisplatin plus fluorouracil (ECF) as the main regimens for 2 to 4 cycles before surgery (Japanese Gastric Cancer Association 2017; Cunningham et al. 2006).

### Definition of BTF

Peri-operative BTF was defined as the transfusion of red blood cells from the admission time to the day of discharge during hospitalization (usually 3–5 days before operation and 10–14 days thereafter). Packed red blood cells were stored in citrate–phosphate–dextrose–adenine anti-coagulant solution whether leukocytes were depleted or not. Although transfusion was performed at the discretion of the healthcare team supervising peri-operative care, the general indication for BTF was the hemoglobin level < 80 g/L. For patients with hemoglobin level between 80 and 100 g/L, BTF was performed based on the risk factors associated with inappropriate oxygenation or hemodynamic unstability (over 65 years, with cardiovascular or respiratory diseases, oxygen consumption, rate of blood loss, and so on) (American Society of Anesthesiologists Task Force on Perioperative Blood Transfusion and Adjuvant Therapies 2006).

The total amount of BTF was classified as low (< 3 U, low-volume BTF group), moderate (3–7.5 U, moderate-volume BTF group) and high volume (> 7.5 U, high-volume BTF group) (Bellantone et al. 1998). According to transfusion timing, BTF was classified into pre-operative (transfusion between admission and surgery, pre-operative BTF group), intra-operative (transfusion during surgery, intra-operative BTF group), and post-operative transfusion (transfusion between surgery and discharge, post-operative BTF group). For those patients who transfused more than one time described above, the first BTF timing was cited for classification.

The type of BTF was classified as leukocyte depleted or leukocyte non-depleted, according to the transfused blood type. The few patients who received both leukocyte-depleted and non-depleted red cells were assigned into the non-depleted BTF group.

### Definition of post-operative infections

The responsible surgeon checked for the development of post-operative morbidity every day during the hospital stay and at every outpatient visit until 30 days after surgery, with morbidity classified into infectious and non-infectious complications. Patients were examined for the presence of any infection according to general surgical practice, clinical symptoms, temperature ≥ 38 °C and/or increased inflammatory biochemical markers (white blood cell count). The diagnosis of different infectious complications (intra-abdominal infection, pneumonia, urinary tract infection, wound infection, and sepsis) was defined according to the Centers for Disease Control and Prevention (Horan and Andrus 2008). Briefly speaking, intra-abdominal infection was defined as an abscess or diffused infection within the abdominal cavity or the presence of an anastomotic leakage. Pneumonia was defined as an infection of the lung based on infiltrate on chest X-ray and/or computed tomography and clinical findings (respiratory symptoms, fever, leukopenia or leukocytosis). Sepsis was defined as the presence of two or more systemic inflammatory response syndrome criteria. Wound infection was defined as deep surgical site infection that required treatment with antibiotics agents or wound drainage. Urinary tract infection was defined according to positive urine culture and clinical findings (fever, urgency, frequency, dysuria, or suprapubic tenderness).

### Data collection

The clinicopathological factors that could potentially influence the likelihood of post-operative outcomes were collected and analyzed. These factors included age, gender, American Society of Anesthesiologists (ASA) score, body mass index (BMI), comorbidities (diabetes mellitus, hypertension, chronic pulmonary/kidney/liver disease, cardiovascular and cerebrovascular disease), pre-operative white blood cell and lymphocyte counts, pre-operative albumin and hemoglobin levels, the type of gastrectomy, operation times, estimated intra-operative blood loss, the pathological TNM stage and so on. Morbidity and mortality data were collected within 30 days post-operatively and classified in accordance with the modified Clavien–Dindo classification (Dindo et al. 2004). Major complications were defined as Clavien–Dindo grade III or greater. For those patients who developed multiple complications, Clavien–Dindo stage was classified according to the most severe one.

### Matching

To minimize the impact of potential selective bias, transfused patients were matched to non-BTF patients using a propensity score described by Rubin et al. (Rubin and Thomas 1996), and was done as previously described (Yang et al. 2016). Propensity scores were based on baseline variables such as age, sex, comorbidities, ASA score, neo-adjuvant chemotherapy, pre-operative white blood cell and lymphocyte count, pre-operative albumin level, operation method, type of resection, combined multi-organ resection, tumor size, tumor location, operation time and the TNM stage. Nearest neighbor matching was performed in a one-to-one ratio without replacement and a caliper width with a 0.01 standard deviation (SD) was specified.

### Statistical analysis

Statistical analyses were performed with IBM SPSS Statistics for Windows (Ver. 24, NY: IBM Corporation). Continuous data are reported as the mean ± SD and comparisons made on normally distributed data using a *t* test. Categorical variables are expressed as numbers and percentages, and were compared by a *χ*^2^ or Fisher exact test. While for ordinal categorical variables, the differences between groups were compared by a rank sum test. Subgroup analysis was performed according to the transfusion timing, amount and leukocyte depletion or not. Risk factors for infection were subjected to univariate analyses using a *χ*^2^ test to assess the effects of each factor. Multivariate logistic regression analysis was carried out for factors with a *P* value ≤ 0.1 after univariate analysis. A *P* value < 0.05 was considered to represent statistical significance.

## Results

### Clinicopathological characteristics of the patients

The clinicopathological characteristics of the 2064 patients are listed in Table 1. There were 1363 males (66.0%) and 701 (34.0%) females, with a mean age of 55.37 ± 10.51 years (range 19–83) and a mean BMI of 21.80 ± 2.97 kg/m^2^ (range 13.84–35.17). 622 patients (30.1%) suffered from one or more types of comorbidities. A total gastrectomy was performed on 482 patients (23.4%), distal subtotal gastrectomy on 1523 (73.8%) patients, and proximal subtotal gastrectomy on 59 patients (2.9%). Laparoscopic or laparoscopy-assisted procedures were performed on 307 patients (14.9%), and 184 patients (8.9%) underwent combined multi-organ resection, including 47 cases of cholecystectomy, 45 cases of partial pancreatectomy, 31 cases of splenectomy and so on. According to the 7th edition of the UICC TNM classification, there were 528 (25.6%) stage I, 434 (21.0%) stage II, and 1102 (53.4%) stage III patients. The mean operation time was 200 ± 54 min (range 70–584), and the mean estimated intra-operative blood loss was 205 ± 120 mL (range 30–2300).

**Table 1:** Clinicopathological characteristics of the entire cohort (*n* = 2064)

Variables	Transfusion group (*n* = 426)	Non-transfusion group (*n* = 1638)	*P* value
Gender (males)	267 (62.3%)	1,096 (66.9%)	0.100
Age (years)	57.91 ± 11.17	54.72 ± 10.24	< 0.001
Body Mass Index (kg/m^2^)	21.95 ± 3.01	21.24 ± 2.78	< 0.001
ASA score			< 0.001
1	53 (12.4%)	261 (15.9%)	
2	286 (67.1%)	1224 (74.7%)	
3	83 (19.5%)	147 (9.0%)	
4	4 (0.9%)	6 (0.4%)	
Smoking history	164 (38.5%)	717 (43.8%)	0.050
Any comorbidities	145 (34.0%)	477 (29.1%)	0.049
History of abdominal surgery	49 (11.5%)	163 (10.0%)	0.347
Neo-adjuvant chemotherapy	27 (6.3%)	98 (6.0%)	0.784
Pre-operative white blood cell count (× 10^9^/L)	5.90 ± 2.00	6.21 ± 1.83	0.002
Pre-operative lymphocyte count (× 10^9^/L)	1.56 ± 0.77	1.86 ± 0.64	< 0.001
Pre-operative albumin (g/L)	35.83 ± 4.89	38.81 ± 4.28	< 0.001
Pre-operative hemoglobin (g/L)	89.62 ± 24.11	126.23 ± 18.26	< 0.001
Complication due to the tumor^a^	203 (47.7%)	252 (15.4%)	< 0.001
Operation method			0.008
Open	380 (89.2%)	1377 (84.1%)	
Laparoscopy	46 (10.8%)	261 (15.9%)	
Type of resection			< 0.001
Subtotal gastrectomy	290 (68.1%)	1292 (78.9%)	
Total gastrectomy	136 (31.9%)	346 (21.1%)	
Combined multi-organ resection	77 (18.1%)	107 (6.5%)	< 0.001
Tumor size (cm)	4.94 ± 2.11	3.89 ± 1.99	< 0.001
Tumor location			< 0.001
Upper	53 (12.4%)	123 (7.5%)	
Middle	114 (26.8%)	316 (19.3%)	
Lower	237 (55.6%)	1148 (70.1%)	
Diffuse	22 (5.2%)	51 (3.1%)	
pTNM stage^b^			< 0.001
I	57 (13.4%)	471 (28.8%)	
II	66 (15.5%)	368 (22.5%)	
III	303 (71.1%)	799 (48.8%)	
Intra-operative blood loss (mL)	261 ± 200	190 ± 83	< 0.001
Operation time (min)	215.75 ± 62.13	196.22 ± 50.87	< 0.001
Post-operative complications classification			< 0.001
None	353 (82.9%)	1,529 (93.3%)	
Infectious complications	51 (12.0%)	78 (4.8%)	
Non-infectious complications	22 (5.2%)	31 (1.9%)	
Post-operative complications severity^c^			< 0.001
None	353 (82.9%)	1529 (93.3%)	
Grade II	45 (10.6%)	80 (4.9%)	
Grade III or greater	28 (6.6%)	29 (1.8%)	
Transferring to ICU post-operation	22 (5.2%)	27 (1.6%)	< 0.001
Post-operative hospital stays (days)	13.47 ± 11.87	11.37 ± 3.70	< 0.001

Data are presented as mean ± SD or *n* (%)

*ASA* American Society of Anesthesiology, *ICU* intensive care unit

^a^Including pyloric obstruction or bleeding

^b^Tumor stages are based on 7th edition of the Union for International Cancer Control TNM classification

^c^Based on the Clavien–Dindo severity classification of surgical complications

426 patients (20.6%) underwent peri-operative BTF and the median amount of blood transfusion was 4 U (range 1.5–42.5). As shown in Table 1, patients receiving BTF were obviously older, with lower pre-operative hemoglobin levels, higher rates of complications due to the tumor (including pyloric obstruction or bleeding), total gastrectomy and/or combined multi-organ resection were performed, at stage III, and had larger tumor sizes and longer operation times.

### Post-operative complications

A total of 376 events occurred in 323 patients of the entire cohort (15.6%). There were 156 events that were classified as grade I complications which occurred in 141 patients (6.8%), including transient vomiting, fever, fluid collection, and/or pain needing antiemetics, antipyretics, diuretics, and/or analgesics to relieve the symptoms (*n* = 94), wound problem treated in bedside (*n* = 31), atelectasis requiring physiotherapy (*n* = 18), and bladder dysfunction requiring urinary catheterization (*n* = 13). Considering their little clinical relevance, grade I complications were not included for further analysis, as reported by Ahmad et al. (2014). The remaining 220 post-operative grade II or greater complications occurred in 182 patients (8.8%), including 139 (63.2%) infections and 81 (36.8%) non-infection complications (Table 2). Among the infection complications, intra-abdominal infection (*n* = 74) was the most common, followed by pneumonia (*n* = 51) and sepsis (*n* = 15). While intestinal obstruction (*n* = 15), pleural effusion (*n* = 12) and ascites (*n * = 11) were ranked as the top three most frequent non-infection complications. According to the Clavien–Dindo classification system, the incidence of stage II, IIIa, IIIb, IVa, IVb and V complications were 6.1% (*n* = 125), 0.9% (*n* = 19), 0.9% (*n* = 19), 0.4% (*n* = 9), 0.05% (*n* = 1) and 0.4% (*n* = 9), respectively. Thus major complications occurred in 57 patients (2.8%). 73 patients (17.1%) in the BTF group developed complications, which were significantly more common than that in the non-BTF group (6.7%, *P* < 0.001), as was the infection rate (12.0% vs 4.8%, *P* < 0.001).

**Table 2:** Post-operative complications of the entire 2064 patients (*n* = 220)

Complications	Number (%)
Infectious	139 (63.2%)
Intra-abdominal infection	74 (33.6%)
Pneumonia	39 (17.7%)
Sepsis not specified	15 (6.8%)
Wound infection	9 (4.1%)
Urinary tract infection	2 (0.9%)
Non-infectious	81 (36.8%)
Intestinal obstruction	15 (6.8%)
Pleural effusion	12 (5.5%)
Ascites	11 (5.0%)
Intra-abdominal bleeding	10 (4.5%)
Lymphatic fistula	7 (3.2%)
Gastrointestinal bleeding	7 (3.2%)
Duodenal stump fistula	3 (1.4%)
Cerebral infarction	3 (1.4%)
Delayed gastric emptying	2 (0.9%)
Anastomotic stricture	2 (0.9%)
Liver failure	2 (0.9%)
Pneumothorax	2 (0.9%)
Cardiac arrest	2 (0.9%)
Diabetic ketoacidosis	1 (0.5%)
Urinary retention	1 (0.5%)
Renal failure	1 (0.5%)

### Propensity score matching analysis

After one-to-one propensity score matching, 361 pairs of patients were enrolled into further analysis. The clinicopathological characteristics of the patients after matching are listed in Table 3. All of the important basic characteristics such as comorbidities, pre-operative white blood cell/lymphocyte counts, the tumor stage and operation times were comparable between the two groups, except for the pre-operative hemoglobin levels (91.19 ± 23.99 g/L vs. 119.98 ± 17.38 g/L, *P* < 0.001) and estimated intra-operative blood loss (248 ± 177 mL vs. 212 ± 90 mL, *P* = 0.001). Sixty patients (16.6%) in the BTF group developed post-operative complications, including 43 cases (11.9%) of infection, which was significantly higher than those in the non-BTF group (9.7% and 6.9%, *P* = 0.006 and *P* = 0.022, respectively).

**Table 3:** Clinicopathological characteristics of the propensity score-matched cohort (*n* = 722)

Variables	Transfusion group (*n* = 361)	Non-transfusion group (*n* = 361)	*P* value
Gender (males)	225 (62.3%)	229 (63.4%)	0.758
Age (years)	57.15 ± 10.92	55.76 ± 11.43	0.096
Body Mass Index (kg/m^2^)	21.26 ± 2.74	21.24 ± 2.77	0.895
ASA score			0.067
1	48 (13.3%)	46 (12.7%)	
2	256 (70.9%)	233 (64.5%)	
3	54 (15.0%)	77 (21.3%)	
4	3 (0.8%)	5 (1.4%)	
Smoking history	136 (37.7%)	125 (34.6%)	0.394
Any comorbidities	115 (31.9%)	119 (33.0%)	0.750
History of abdominal surgery	40 (11.1%)	37 (10.2%)	0.718
Neo-adjuvant chemotherapy	26 (7.2%)	16 (4.4%)	0.112
Pre-operative white blood cell count (× 10^9^/L)	5.86 ± 1.87	6.10 ± 1.73	0.080
Pre-operative lymphocyte count (× 10^9^/L)	1.62 ± 0.74	1.66 ± 0.65	0.416
Pre-operative albumin (g/L)	36.39 ± 4.87	36.91 ± 4.96	0.154
Pre-operative hemoglobin (g/L)	91.19 ± 23.99	119.98 ± 17.38	< 0.001
Complication due to the tumor^a^	147 (40.7%)	147 (40.7%)	1.000
Operation method			0.091
Open	322 (89.2%)	335 (92.8%)	
Laparoscopy	39 (10.8%)	26 (7.2%)	
Type of resection			0.203
Subtotal gastrectomy	260 (72.0%)	275 (76.2%)	
Total gastrectomy	101 (28.0%)	86 (23.8%)	
Combined multi-organ resection	52 (14.4%)	50 (13.9%)	0.831
Tumor size (cm)	4.77 ± 2.11	4.86 ± 2.02	0.544
Tumor location			0.441
Upper	43 (11.9%)	40 (11.1%)	
Middle	92 (25.5%)	75 (20.8%)	
Lower	210 (58.2%)	228 (63.2%)	
Diffuse	16 (4.4%)	18 (5.0%)	
pTNM stage^b^			0.977
I	55 (15.2%)	43 (11.9%)	
II	58 (16.1%)	74 (20.5%)	
III	248 (68.7%)	244 (67.6%)	
Intra-operative blood loss (mL)	248 ± 177	212 ± 90	0.001
Operation time (min)	211.78 ± 58.61	216.61 ± 45.50	0.199
Post-operative complication classification			0.023
None	301 (83.4%)	326 (90.3%)	
Infectious complications	43 (11.9%)	25 (6.9%)	
Non-infectious complications	17 (4.7%)	10 (2.8%)	
Post-operative complication severity^c^			0.006
None	301 (83.4%)	326 (90.3%)	
Grade II	38 (10.5%)	23 (6.4%)	
Grade III or greater	22 (6.1%)	12 (3.3%)	
Transferring to ICU post-operation	16 (4.4%)	11 (3.0%)	0.327
Post-operative hospital stays (days)	13.20 ± 10.70	12.34 ± 4.05	0.153

Data are presented as mean ± SD or *n* (%)

*ASA* American Society of Anesthesiology, *ICU* intensive care unit

^a^Including pyloric obstruction or bleeding

^b^Tumor stages are based on 7th edition of the Union for International Cancer Control TNM classification

^c^Based on the Clavien–Dindo severity classification of surgical complications

### Subgroup analysis

Of the 361 patients receiving BTF who were enrolled into further analysis after matching, the median amount of transfused blood was 4 U (range 1.5–42.5). The transfusion timing is shown in detail in Table 4a. There were 149 patients who received transfusion pre-operatively, 128 intra-operatively and 189 post-operatively. The incidence of infection in the intra-operative BTF group was 14.1%, and was significantly higher than in the non-BTF and post-operative BTF groups (6.9 and 6.9%, *P* = 0.014 and *P* = 0.035, respectively). However, the infection rates among the non-BTF, pre-operative and post-operative BTF groups were not statistically different. There were 95 patients who received BTF more than once in the period described above, of whom 15 (15.8%) developed infections, which was significantly higher than that in the non-BTF group (6.9%, *P* = 0.007), but comparable with those in the only one time period transfused patients (10.5%, *P* = 0.174).

**Table 4:** Subgroup analysis after matching (*n* = 722)

Variables	Complications	
Transfusion timing	None	Infections
a. Transfusion timing and infections		
Non-BTF group (*n* = 361)	336 (93.1%)	25 (6.9%)
Pre-operative BTF group (*n* = 149)	136 (91.3%)	13 (8.7%)
Intra-operative BTF group (*n* = 128)	110 (85.9%)	18 (14.1%)
Post-operative BTF group (*n* = 189)	176 (93.1%)	13 (6.9%)
One time-period BTF group (*n* = 266)	238 (89.5%)	28 (10.5%)
Multi-time-period BTF group (*n* = 95)	80 (84.2%)	15 (15.8%)
Comparison between subgroups: non-BTF vs. pre-operative BTF, *P* = 0.482; non-BTF vs. intra-operative BTF, ***P*** **= 0.014**; non-BTF vs. post-operative BTF, *P* = 0.984; pre-operative BTF vs. intra-operative BTF, *P* = 0.160; pre-operative BTF vs. post-operative BTF, *P* = 0.527; intra-operative BTF vs. post-operative BTF, ***P*** **= 0.035**		
Non-BTF vs. one time-period BTF, *P* = 0.109; non-BTF vs. multi-time-period BTF, ***P*** **= 0.007**; one time-period BTF vs. multi-time-period BTF, *P* = 0.174		

Variables	Complications	
Transfusion volume	None	Infections
b. Transfusion volume and infections		
Non-BTF group (*n* = 361)	336 (93.1%)	25 (6.9%)
Low-volume BTF group (*n* = 74)	66 (89.2%)	8 (10.8%)
Moderate-volume BTF group (*n* = 228)	205 (89.9%)	23 (10.1%)
High-volume BTF group (*n* = 59)	47 (79.7%)	12 (20.3%)
Comparison between subgroups: non-BTF vs. low-volume BTF, *P* = 0.250; non-BTF vs. moderate-volume BTF, *P* = 0.172; non-BTF vs. high-volume BTF, ***P*** **= 0.001**; low-volume BTF vs. moderate-volume BTF, *P* = 0.859; low-volume BTF vs. high-volume BTF, *P* = 0.127; moderate-volume BTF vs. high-volume BTF, ***P*** **= 0.032**		

Variables	Complications	
Transfusion type	None	Infections
c. Leukocyte depletion and infections		
Non-BTF group (*n* = 361)	336 (93.1%)	25 (6.9%)
Leukocyte-depleted BTF group (*n* = 221)	197 (89.1%)	24 (10.9%)
Leukocyte-non-depleted BTF group (*n* = 140)	121 (86.4%)	19 (13.6%)
*BTF* blood transfusion		
Comparison between subgroups: non-BTF vs. leukocyte-depleted BTF, *P* = 0.097; non-BTF vs. leukocyte-non-depleted BTF, ***P*** **= 0.018**; leukocyte-depleted BTF vs. leukocyte-non-depleted BTF, *P* = 0.438		

Statistically significant values are in bold (*p* < 0.05)

74 patients (20.5%) received a low-volume (< 3 U), 228 (63.2%) a moderate-volume (3-7.5 U), and 59 (16.3%) a high-volume (> 7.5 U) BTF, respectively (Table 4b). 12 patients (20.3%) in the high-volume BTF group suffered from post-operative infections, which were significantly higher than those in the non-BTF group (6.9%, *P* = 0.001), and moderate-volume BTF group (10.1%, *P* = 0.032). In contrast, the infection rates among the non-BTF, low-volume and moderate-volume BTF groups were not significantly different.

221 patients (61.2%) received BTF with leukocyte depletion and the remaining 140 were transfused with leukocyte-non-depleted blood (Table 4c). Patients receiving leukocyte-non-depleted BTF had a higher risk of infection compared with those not receiving BTF (13.6 and 6.9%, *P* = 0.018). Patients who were transfused with leukocyte-depleted blood appeared to have a trend toward a higher incidence of infection compared with those not receiving BTF (10.9 vs 6.9%), but the difference was not statistically significant (*P* = 0.097). There was no significant difference between the incidence of infections among patients who received BTF with leukocyte-depleted or non-depleted blood (*P* = 0.438).

### Univariate and multivariate logistic regression analysis

On univariate analysis (Table 5), combined multi-organ resection, operation time ≥ 240 min, BMI ≥ 25 kg/m^2^, a history of smoking, splenectomy and BTF were clarified as risk factors for infection in the 722 matched patients. After multivariate analysis (Table 6), including factors that had *P* values ≤ 0.1 established by univariate analysis, operation time ≥ 240 min (odds ratio, OR = 2.378, 95% confidence interval, CI 1.393–4.062, *P* = 0.002), combined multi-organ resection (OR = 2.418, 95% CI 1.281–4.561, *P* = 0.006), BTF (OR = 1.872, 95% CI 1.094–3.204, *P* = 0.022) and BMI ≥ 25 kg/m^2^ (OR = 2.149, 95% CI 1.074–4.299, *P* = 0.031) were shown to be independent risk factors for infection. Smoking appeared to influence the incidence of infection (OR = 1.597, 95% CI 0.945–2.699), but the finding was not statistically significant (*P* = 0.080). No other variables such as comorbidities, total gastrectomy or splenectomy were identified as independent risk factors for infection.

**Table 5:** Univariate analysis of possible predictors for post-operative infections following radical gastrectomy for gastric cancer after matching (*n* = 722)

Variables	Infections (*n* = 68)	Non-infection (*n* = 654)	*χ*^2^ value	*P* value
Sex (male: female)	48/20	406/248	1.911	0.167
Age (years) ≥ 65/<65	20/48	158/496	0.915	0.339
BMI (kg/m^2^) ≥ 25/<25	13/55	60/594	6.701	0.010
Smoking history; yes/no	34/34	227/427	6.239	0.012
ASA score ≥ 3/<3	16/52	123/531	0.883	0.347
Comorbidity; yes/no	28/40	206/448	2.634	0.105
Pre-operative white blood cell count (× 10^9^/L) ≥ 4/<4	64/4	584/70	1.556	0.212
Pre-operative lymphocyte count (× 10^9^/L) ≥ 1.5/<1.5	38/30	353/301	0.090	0.764
Pre-operative hemoglobin (g/L) ≥ 100/<100	43/25	389/265	0.361	0.548
Pre-operative albumin (g/L) ≥ 35/ <35	39/29	429/225	1.836	0.175
Complication due to the tumor^a^; yes/no	28/40	266/388	0.006	0.936
Neo-adjuvant chemotherapy; yes/no	4/64	38/616	0.001	0.981
Operation method; open/laparoscopy	7/61	58/596	0.153	0.696
Extent of gastric resection; subtotal/total	48/20	487/167	0.482	0.487
Combined multi-organ resection; yes/no	22/46	80/574	20.555	< 0.001
Splenectomy; yes/no	5/63	11/643	0.141	0.002
Operation time (min) ≥ 240/< 240	36/32	183/471	18.159	< 0.001
Intra-operative blood loss (mL) ≥ 300/<300	26/42	191/463	2.389	0.122
Tumor size (cm) ≥ 5/<5	39/29	343/311	0.595	0.440
Depth of invasion; T4/T1–3	55/13	489/165	1.239	0.266
Lymph node metastasis; positive/negative	48/20	469/185	0.038	0.845
pTNM stage; III/I–II	49/19	433/221	0.950	0.330
Peri-operative BTF; yes/no	43/25	318/336	5.260	0.022

*BMI* body mass index, *ASA* American Society of Anesthesiologist, *BTF* blood transfusion

^a^Including pyloric obstruction or bleeding

**Table 6:** Multivariate analysis of possible predictors for post-operative infections following gastrectomy for gastric cancer after matching (*n* = 722)

Variables	Odds ratio (OR)	95% confidence interval (CI)	*P* value
Operation time ≥ 240 min	2.378	1.393–4.062	0.002
Combined multi-organ resection	2.418	1.281–4.561	0.006
Peri-operative blood transfusion	1.872	1.094–3.204	0.022
Body mass index ≥ 25 kg/m^2^	2.149	1.074–4.299	0.031
Smoking history	1.597	0.945–2.699	0.080
Splenectomy	1.555	0.446–5.418	0.480

## Discussion

In this retrospective study of a large cohort of patients from a single high-volume center in China, after one-to-one propensity score matching we found that infection was the most common complication following radical gastrectomy for gastric cancer, leading to prolonged post-operative hospital stays (23.78 vs. 11.62 days, *P* < 0.001) and a higher frequency of requiring intensive care (17.6 vs 2.3%, *P* < 0.001) and also mortality (8.8 vs 0.2%, *P* < 0.001). Thus, surgeons should prioritize operating procedures to reduce the incidence of infection. Although many studies have evaluated the impact of peri-operative BTF on post-operative outcomes of patients undergoing gastrectomy for gastric cancer, unfortunately most of the conclusions were based on a limited number of patients and unmatched analysis. Thus, the conclusions may be confused by other factors such as comorbidities and more extended resections. Propensity score matching analysis is widely used in retrospective cohort studies to control for confounding biases, mimicking a randomized trial, with the assumption that all related confounders are controlled (Fujiya et al. 2016). As listed in Table 1, background data between the patients receiving BTF or not were significantly different before matching and thus it could be argued that direct comparison of the infection rate is not appropriate because some of the basic data could be independently responsible for infections, regardless of BTF. To the best of our knowledge, this is the first study to investigate the relationship between BTF and post-operative infections that has used propensity score-matching analysis to adjust for basic data between patients with or without BTF following gastrectomy for gastric cancer. After matching, most of the important background data become comparable except for pre-operative hemoglobin levels and intra-operative blood loss, which were considered to be the main factors associated with BTF. These data were not used for enrolment for matching to avoid too many patients who received BTF being excluded because of a lack of matching. Further univariate and multivariate analyses showed that pre-operative hemoglobin anemia (< 100 g/L) and intra-operative blood loss ≥ 300 mL were not associated with increased risk of post-operative infection.

The significance of the transfusion timing and volume, and impact of leukocyte depletion on post-operative infections have rarely been reported. By sub-group analysis, patients who received intra-operative BTF were identified as being at the highest risk of developing an infection, while pre-operative and post-operative BTF showed less impact. Possible explanations include that intra-operative BTF is usually performed because of massive bleeding, even hemorrhagic shock in patients with extended resection or iatrogenic injury, which may have increased the risk of infection. Intra-operative BTF may also act synergistically with surgical stress to induce immunosuppression (Kanda et al. 2016). Thus, for patients who suffered from anemia at admission, performing BTF pre-operatively instead of intra-operatively may decrease the risk of infection. Additionally, for patients with mild to moderate bleeding during surgery, given good hemodynamic stability, there could be some room for consideration of giving BTF post-operatively instead of intra-operatively. But the most suitable time interval between surgery and BTF is hard to define and requires further prospective investigation. Another interesting finding was that patients who received BTF in more than one time period, classified as pre-, intra- and post-operative, had a significantly higher risk of developing infection compared with those receiving BTF for only one time period, reminding us that we should avoid performing BTF in multi-time periods if possible. The potential cause may be because of the prolonged process of immunosuppression induced by BTF but the exact reasons remain to be elucidated.

The volume of BTF on post-operative outcomes remains controversial; some studies clarified that intra-operative transfusion of even 1–2 U of packed red blood cells was associated with increased 30-day mortality, surgical site infection, pneumonia and sepsis in general surgery patients, whereas others declared that only a large volume of BTF increased the risk (Bellantone et al. 1998; Bernard et al. 2009; Ferraris et al. 2012; Xiao et al. 2014). In the present study, only patients who were given a high-volume (> 7.5 U) BTF showed an increased risk of infection, while administration of a low and moderate volume showed no significant impact. It should be borne in mind that patients who required large-volume BTF, always suffer from severe anemia or massive bleeding, meaning a worse physical condition and a higher risk of post-operative complications.

Allogeneic leukocytes are assumed to play a critical role in inducing TRIM and significantly affect the post-operative outcomes in various types of surgery (Cervia et al. 2007; Tartter et al. 1998). There has been a randomized trial concluded that both the incidence of the operative site and nosocomial infections were significantly higher in patients transfused with packed red cells than those in patients transfused with leukocyte-depleted red cells; but there were only 13 stomach procedures (Tartter et al. 1998). In our hospital, patients who were given BTF received red blood cells without leukocyte depletion before 2014, with depletion after then, giving us the opportunity to evaluate the impact of leukocyte depletion on the incidence of infection directly. As shown in Table 4c, patients who received a leukocyte non-depleted BTF had a significantly higher risk of developing an infection compared to those not receiving a BTF (13.6 vs 6.9%, *P* = 0.018), while receiving leukocyte-depleted BTF seemed not to increase the risk (10.9%, *P* = 0.097). The present study supports the hypothesis that susceptibility to infection following BTF is due to leukocytes in the transfused blood, and clearly supports the use of leukocyte-depleted blood in patients undergoing gastrectomy for gastric caner who require a BTF.

In consistent with our previous study and similar studies from other institutes, a longer operative time (≥ 240 min), combined multi-organ resection and being overweight (BMI ≥ 25 kg/m^2^) were identified as independent risk factors for post-operative infection following gastrectomy for gastric cancer (Xiao et al. 2017; Brar et al. 2012; Hirao et al. 2013; Lee et al. 2014). It is easy to understand why and has been discussed in our previous study (Xiao et al. 2017). In contrast to the conclusions of the present investigation, peri-operative BTF was not identified as an independent risk factor for intra-abdominal infections (IAI) in our previous study. While BTF has been identified as an independent risk factor for pneumonia and sepsis after upper gastrointestinal cancer resections in several previous studies (Aquina et al. 2015; Miki et al. 2016). To explore the underlining reasons, we further divided infections into local infections (including IAI and wound infection) and systemic infections (including pulmonary infection, sepsis and urinary tract infection). Among the matched 361 patients who were not given BTF, 25 patients developed post-operative infection, including 18 cases of local infections and 9 cases of systemic infections (2 patients developed both local and systemic infections); while in the 43 patients who developed infection among the 361 patients receiving BTF, there were 22 cases of local and 24 cases of systemic infections (3 patients developed both local infections and systemic infections). The incidence of local infections were similar in the patients receiving BTF or did not (6.1 vs 5.0%, *P* = 0.515), but systemic infection was significantly more common in the BTF group compared to the non-BTF group of patients (6.6 vs 2.5%, *P* = 0.008). Thus, BTF may mainly increase the risk of systemic infection but has a limited influence on the occurrence of local infection. A possible explanation is that local infections are largely dependent on surgical procedures, whereas systemic infections are mainly associated with systemic inflammation and immunity, but the exact underlying mechanisms remain unclear.

As reported in other studies, peri-operative BTF was clarified as a predictor for infection by multivariate analysis after matching in the present study (Squires et al. 2015; Jung et al. 2013; Brar et al. 2012). By carefully reviewing the medical records, we found that there were eight patients who received BTF simultaneously or after infection, but without any clinical evidence of bleeding. A low-hemoglobin level and hemodilution, as a result of extra-cellular expansion during the stress response in patients who developed severe infections, may act as a confounder for the association between BTF and the complications of infection (Bellantone et al. 1998). As listed in Table 3, 43 patients (11.9%) who received BTF developed an infection, which was significantly more common than in those not receiving a BTF (25 cases, 6.9%, *P* = 0.022). But if we ruled out the eight pairs of patients, the incidence of infection was comparable between the patients receiving BTF or not (9.9 vs 7.1%, *P* = 0.177). Maybe it is not BTF itself but the circumstances necessitating BTF that are the real determinants of outcomes (Bellantone et al. 1998), although a prospective international multi-center study with a larger sample size will be needed to confirm this conjecture.

There are several limitations of the present study including its retrospective nature, single-institution design and insufficient data on immune functions. In addition, some patients in the present study received platelet or plasma transfusions, which may also affect the patients’ immune status or interact with BTF to influence post-operative outcomes; we did not investigate these potential associations (Xie et al. 2015; Subramanian et al. 2012). Last but not the least, although propensity score-matching analysis has the advantage of reducing selective bias, it restricts the analysis to a relatively small proportion of the patients, thus dramatically increases the possibility of a type II error and limits the statistical power. However, the prospectively registered high-volume sample database that collected and stored detailed data, the combination use of propensity score matching and multivariate analyses can offer more powerful statistical strength and make our final conclusions to be more robust and reliable. Given it would be unethical to randomize patients to perform BTF or not, large sample-based observational analysis is the best alternative to investigate the effects of BTF on the post-operative infection rate.

In conclusion, the propensity score-matched analysis from a high-volume center in China revealed that infection was the most common complication following gastrectomy for gastric cancer, leading to prolonged hospitalization, a higher frequency of requiring intensive care and also mortality. Peri-operative BTF was identified as an independent risk factor for developing infection, especially a systemic infection such as pneumonia. Patients who were given high-volume (> 7.5 U) leukocyte non-depleted BTF and transfused intra-operatively seemed to be at the highest risk. But it should be remembered that the real relationship between BTF and infection maybe confused by the chronological order and further prospective studies are definitely warranted.
